# The Effects of Repetitive Transcranial Magnetic Stimulation on Cognitive Impairment and the Brain Lipidome in a Cuprizone-Induced Mouse Model of Demyelination

**DOI:** 10.3389/fnins.2021.706786

**Published:** 2021-07-14

**Authors:** Cuihong Zhou, Min Cai, Ying Wang, Wenjun Wu, Yuezhen Yin, Xianli Wang, Guangtao Hu, Huaning Wang, Qingrong Tan, Zhengwu Peng

**Affiliations:** ^1^Department of Psychiatry, Xijing Hospital, Fourth Military Medical University, Xi’an, China; ^2^Shaanxi Key Lab of Free Radical Biology and Medicine, The Ministry of Education Key Lab of Hazard Assessment and Control in Special Operational Environment, Department of Toxicology, School of Public Health, Fourth Military Medical University, Xi’an, China; ^3^Minkang Hospital, Ningxia Hui Autonomous Region, Yinchuan, China; ^4^Department of Psychiatry, Southwest Hospital, Army Medical University, Chongqing, China

**Keywords:** brain lipidome, cuprizone, demyelination, rTMS, cognitive impairment

## Abstract

The protective effects of repetitive transcranial magnetic stimulation (rTMS) on myelin integrity have been extensively studied, and growing evidence suggests that rTMS is beneficial in improving cognitive functions and promoting myelin repair. However, the association between cognitive improvement due to rTMS and changes in brain lipids remains elusive. In this study, we used the Y-maze and 3-chamber tests, as well as a mass spectrometry-based lipidomic approach in a CPZ-induced demyelination model in mice to assess the protective effects of rTMS on cuprizone (CPZ)-induced cognitive impairment and evaluate changes in lipid composition in the hippocampus, prefrontal cortex, and striatum. We found that CPZ induced cognitive impairment and remarkable changes in brain lipids, specifically in glycerophospholipids. Moreover, the changes in lipids within the prefrontal cortex were more extensive, compared to those observed in the hippocampus and striatum. Notably, rTMS ameliorated CPZ-induced cognitive impairment and partially normalized CPZ-induced lipid changes. Taken together, our data suggest that rTMS may reverse cognitive behavioral changes caused by CPZ-induced demyelination by modulating the brain lipidome, providing new insights into the therapeutic mechanism of rTMS.

## Introduction

Repetitive transcranial magnetic stimulation (rTMS) is a non-invasive intervention widely used to treat several psychiatric disorders, such as major depressive disorder (MDD) (Miron et al., 2019), posttraumatic stress disorder (PTSD) (Philip et al., 2019) and schizophrenia (Jiang et al., 2019). White matter integrity and function is a predictor for the treatment response of rTMS for PTSD and MDD (Barredo et al., 2019), and low-intensity TMS promote the survival and maturation of newborn oligodendrocytes in the adult mouse brain (Cullen et al., 2019). Moreover, rTMS has also been shown to be beneficial in treating demyelinating disorders, such as Alzheimer’s disease and multiple sclerosis (San et al., 2019; Chou et al., 2020); thus, the protective effects of myelin might be involved in the beneficial effects of rTMS.

Lipids play a key role in membrane composition, energy metabolism, neurotransmission, and neuromodulation of multiple molecular pathways (Piomelli, 2005; Brown and Murphy, 2009), and substantially influence biological processes associated with learning, memory, and the regulation of emotional behavior (Hashimoto et al., 2015; McDougall et al., 2017). The involvement of lipids in modulating synaptic physiology, receptor pharmacology, energy provision, and metabolism in the brain has been well clarified (Egawa et al., 2016; Araque et al., 2017; Wu et al., 2018). The extent of myelination is correlated with brain structure and function, and the regulation of myelination is critical for various neurophysiological functions (Almeida and Lyons, 2017; Monje, 2018; Williamson and Lyons, 2018; Hussain et al., 2019). The role of myelination in cognitive function has been well studied (Geraghty et al., 2019; Kato et al., 2020), and decreased myelin has been implicated in cognitive deficits in various neurological disorders, such as schizophrenia, MDD, and Alzheimer’s disease (Bartzokis et al., 2007; Haroutunian et al., 2014; Li et al., 2016). Myelin is composed of compact lipid membranes that wrap around axons, providing trophic support, as well as electrical insulation to aid the efficient propagation of action potentials. Diseases affecting myelin are associated with alterations in its lipid composition (Schmitt et al., 2015; Maganti et al., 2019).

Notably, our recent study found that the modulation of the brain lipidome might be involved in the antidepressant action of rTMS (Xue et al., 2020). However, little is known about the influence of rTMS on demyelination-induced cognitive impairment and the concomitant changes in brain lipids.

The cuprizone (CPZ) mouse model allows the investigation of the complex molecular mechanisms behind non-autoimmune-mediated demyelination and spontaneous remyelination, which can be used as a model for multiple sclerosis and schizophrenia (Praet et al., 2014; Wang H.N. et al., 2015). Furthermore, the hippocampus, PFC, and striatum are interconnected brain regions that play central roles in higher brain functions, including learning, memory, and the planning of complex cognitive behaviors (Goncalves et al., 2016; Hiser and Koenigs, 2018; Corbit et al., 2019). Dysfunction in the circuitry containing these regions has been described in multiple psychiatric disorders (Henseler et al., 2010; Lanz et al., 2019). CPZ causes cognitive impairment, demyelination, and alterations in the lipid composition in the PFC, hippocampus, and striatum (Nickel et al., 2019; Nomura et al., 2019; Zhou et al., 2020). Thus, in this study, we employed a CPZ-induced mouse model of demyelination and used lipidomic analysis to investigate the effects of rTMS on CPZ-induced cognitive impairment and the associated lipid changes after treatment for 7 days. We found that rTMS improved cognitive behaviors in CPZ-induced mice and caused changes in various lipid species in the hippocampus, PFC, and striatum, indicating that the re-myelination effect of rTMS might be caused by a region-specific regulation of the brain lipidome.

## Materials and Methods

### Animals

Adult male C57BL/6 mice (8 weeks old, weighing 18–22 g) were group-housed (four per cage) in cages at 20–25°C and maintained on a 12 h light/dark daily cycle (lights on from 8 a.m. to 8 p.m.) with *ad libitum* access to food and water. All animal experiments in this study were approved by the Animal Use and Protection Committee of the Fourth Military Medical University and carried out in accordance with the National Institutes of Health Guide for the Care and Use of Laboratory Animals.

### Experimental Design

**FIGURE 1:**
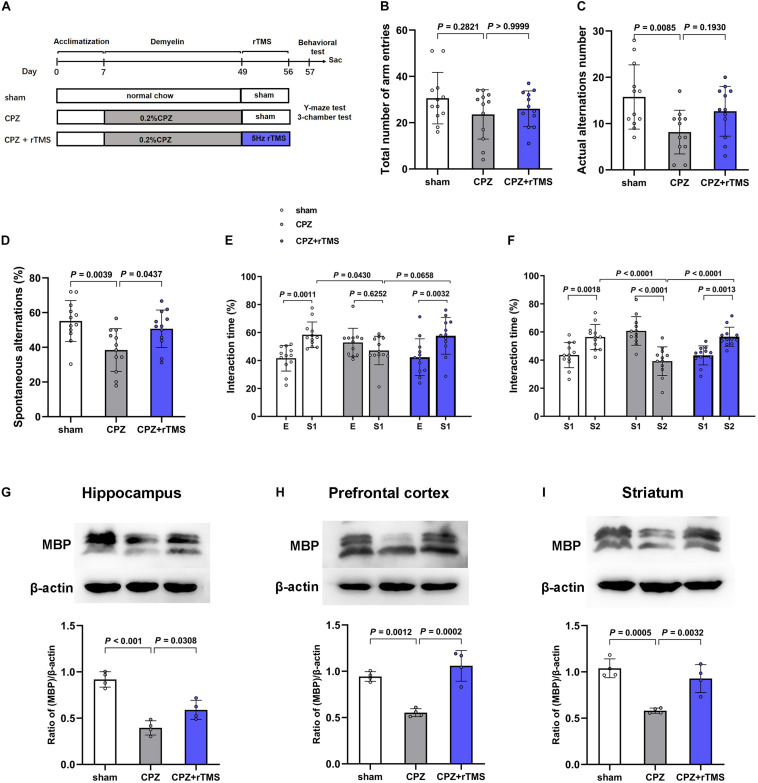
rTMS ameliorated CPZ-induced cognitive impairment and demyelination in mice. **(A)** Schematic diagram displaying the time course of rTMS treatment in a CPZ-induced mouse model of demyelination. C57BL/6 mice were administered CPZ for 6 weeks to induce demyelination, and rTMS was administered for 1 week after the conclusion of CPZ administration. **(B–D)** In the Y-maze test, CPZ-treated mice showed impaired spatial working memory and spatial cognitive ability, which was restored by rTMS. **(E,F)** In the 3-chamber test, mice in the CPZ group showed reduced social interactions and weaker social memory. Mice in the CPZ + rTMS group performed better than those treated with CPZ alone. **(G–I)** Representative immunoblots and densitometry analysis of MBP in the hippocampus, PFC, and striatum in the sham, CPZ, and CPZ + rTMS groups. E, empty wire-cup; S1, subject 1; S2, subject 2.

After 7 days of acclimatization, 36 mice were randomly assigned to three groups (12 per group): Sham, CPZ, and CPZ + rTMS (Figure 1A). Mice in the CPZ group were administered CPZ, 0.2% by weight, in standard powdered rodent chow for 6 weeks, then fed regular chow, and treated with sham rTMS for 7 days; concurrently, mice in the sham group were fed a normal powdered rodent chow and treated with sham rTMS; mice in the CPZ + rTMS group were administered CPZ, 0.2% by weight, in standard powdered rodent chow for 6 weeks, then fed regular chow, and treated with rTMS (5 Hz, 1.26 T) for 7 days. Twenty-four hours after the treatment, all the mice were subjected to the Y-maze and 3-chamber tests. Then, the mice were sacrificed, and the PFC, striatum, and hippocampus were immediately collected in liquid nitrogen for later lipidomic analysis by high performance liquid chromatography–mass spectrometry and western blotting.

### CPZ Administration

Based on previous studies (Xiao et al., 2008; Wang H. et al., 2015), CPZ (bis-cyclohexanone oxaldihydrazone) was purchased from Sigma-Aldrich (St. Louis, MO, United States) and mixed into the ground standard rodent chow in a concentration of 0.2% by weight.

### rTMS Treatment

rTMS was administered with a circular coil (inner diameter, 2.5 cm; outer diameter, 5 cm; custom-made YIRD, China). The daily stimulation consisted of 100 trains of 10 pulses delivered at 5 Hz with a 1 s inter-train interval, and the intensity of stimulation was 1.26 T. The rTMS trains were administered daily for 7 days at a rate of 1,000 pulses per day. In the CPZ + rTMS group, the center of the coil was placed over the vertex of the skull, with the coil handle parallel to the axis of the animal’s vertebral column. In the other two groups (sham TMS), the coil was held 10 cm above and separated from the animals’ heads using a 3 cm plastic spacer cube to ensure that they felt the vibrations produced by the click of the coil without any brain stimulation. The movement of the mice was manually restricted during stimulation; therefore, to exclude putative effects of non-specific stress, each animal was allowed to adapt to the rTMS artifact noise and subjected to a daily sham stimulation 5 min per day for 1 week. The real and sham rTMS did not produce seizures or any behavioral changes throughout the treatment period.

### Behavioral Testing

Behavioral testing was performed 24 h after the last rTMS. The mice were brought to the testing area 2 h before the start of all tests. The tests were videotaped and scored by a trained observer. All tests were conducted under low light conditions between 2 p.m. and 6 p.m. The test area was cleaned with 75% ethanol between tests to prevent odor interference and cross infection. The Y-maze test is used to measure the exploration of novelty which infers potential deficits in spatial memory function, using a three arm “maze.” Reduced exploration/alteration frequency of entry into the arms indicates declining cognitive function. The test apparatus consists of three arms (A,B, and C) (30 cm × 10 cm) positioned at equal angles from each other (120°) and surrounded by various extra-maze cues elevated 45 cm above the floor. The mice were randomly placed on one arm and allowed to explore all three arms freely for 8 min. The entire exploration process was videotaped, and the number and order of the exploration of each arm were recorded. Spontaneous alternation (SA) was defined as the tendency to explore new arms over the one previously chosen. The SA rate was calculated as the percentage, as follows: SA percentage = (number of actual alternations)/(total number of arm entries–2) × 100. A reduced SA rate indicated declining cognitive function (Makinodan et al., 2009).

The 3-chamber test is used to measure social motivation and the social interaction, animals are allowed to meet wire-cup, familiar or unfamiliar strange animals in a free environment, the time spent in sniffing each target are scored as a measure of social interaction for rodents. The test was performed in a rectangular box composed of three connected chambers of the same size (30 cm × 30 cm × 30 cm) (Faizi et al., 2011; Kaidanovich-Beilin et al., 2011). A wire cup-like container with a removable lid was fixed in the middle of each side chamber. The experiment consisted of three consecutive 10-min sessions with no inter-trial interval, behavior was measured for 10 min in each session. In the first “habituation” session, subject mice were allowed to freely investigate the 3-chamber box, In session 2, one strange mouse (subject 1) of the same background, age, gender, and weight as the subject mouse was placed inside a wire cup-like container. The subject mouse was placed in the middle chamber for 5 min before being given access to the side chambers for 10 min. The time spent by the subject mice in each chamber was videotaped. In session 3, another unfamiliar mouse (subject 2, also of the same background, age, gender, and weight as the subject mouse) was placed in the previously empty container, and subject 1 was placed in the other chamber. The time spent in each chamber was calculated, and the percentage of interaction time was calculated as the ratio of interaction time with one chamber to the total interaction time with both chambers during the first 5 min of each session (Ueda et al., 2021). The stranger mice (subject 1 and 2) had no encounters with the subject mice prior to testing.

### Tissue Collection

The mice were euthanized by cervical dislocation 24 h after the last intervention, and their brains were removed and rinsed with phosphate-buffered saline. The PFC were isolated in a brain tank (68713, RWD, Shenzhen, China), striatum and hippocampus were isolated under anatomic microscope. All tissues were cut up on ice and weighed. 8 brain tissues of each group were frozen in liquid nitrogen, and stored for later lipidomic analysis by high performance liquid chromatography–mass spectrometry, another 4 brain tissues in each group were lysed in protein extract buffer for later western blot.

### Sample Extraction

We extracted lipids using methyl tert-butyl ether as described previously (Jiang et al., 2017). Briefly, we homogenized the samples (30 mg) with 200 μL of water and 240 μL of methanol. Then, we added 800 μL of methyl tert-butyl ether, subjected the samples to ultrasonication for 20 min at 4°C, and allowed the samples to rest for 30 min at room temperature. To separate the organic components, we centrifuged the solution at 10°C for 15 min (14,000 × g). We collected the upper aqueous layer (containing the organic solvent phase), evaporated the solvent under nitrogen, and stored it at –80°C until use.

### Lipid Analysis by Liquid Chromatography–Mass Spectrometry

We selected reverse phase chromatography for separation by liquid chromatography with a CSH C18 column (1.7 μm, 2.1 mm × 100 mm, Waters). The lipid extracts were redissolved in 200 μL of 90% isopropanol/acetonitrile and centrifuged at 14,000 × g for 15 min; finally, 3 μL of each sample was injected for analysis. Solvent A was acetonitrile-water (6:4, v/v) with 0.1% formic acid and 0.1 mM ammonium formate; solvent B was acetonitrile–isopropanol (1:9, v/v) with 0.1% formic acid and 0.1 mM ammonium formate. The initial mobile phase was 30% solvent B at a flow rate of 300 μL/min, which was held for two min, then linearly increased to 100% solvent B for 23 min, followed by equilibration in 5% solvent B for 10 min. The autosampler was maintained at 10°C. To avoid the influence of instrumentation error, signal fluctuations were detected in a random order, even during sample analysis. We performed sampling of the queue every eight samples using one of the quality control samples to monitor and evaluate the reliability of the experimental data.

After ultra-high performance liquid chromatography separation, we analyzed the samples using a Q Exactive^TM^ plus mass spectrometer. The parameters for the positive ion mode were as follows: Heater temperature, 300°C; sheath gas flow rate, 45 arb; auxiliary gas flow rate, 15 arb; sweep gas flow rate, 1 arb; spray voltage, 3.0 kV; capillary temperature, 350°C; S-Lens radiofrequency level, 50%; and MS1 scan range, 200–1,800 m/z. For the negative ion mode, the parameters were as follows: Heater temperature, 300°C; sheath gas flow rate, 45 arb; auxiliary gas flow rate, 15 arb; sweep gas flow rate, 1 arb; spray voltage, 2.5 kV; capillary temperature, 350°C; S-Lens radiofrequency level, 60%; and MS2 scan range, 250–1,800 m/z.

### Lipid Identification Using LipidSearch^TM^

LipidSearch^TM^ is a search engine used to identify lipid species based on mass spectroscopy data. It contains data on >30 lipid classes and >1,500,000 ion fragments. Mass tolerances for both molecular precursors and fragment ions were set to 5 ppm. The displayed product ion threshold was set at five, and grades A, B, C, and D were all used in the identification quality filtering. All lipid classes in the database, including 71 sub-species, were chosen for identification. Adducts of + H, + NH4 were selected for positive mode searches, and −H, + CH3COO were selected for negative mode searches since ammonium acetate was used in the mobile phases.

### Western Blot Analysis

The brain tissues (PFC, striatum, and hippocampus) were cut into pieces, then, weighed and lysed (62.5 mM Tris-HCl, 2% w/v sodium dodecyl sulfate, 10% glycerol, 50 mM dithiothreitol, and 0.1% w/v bromophenol blue). The protein concentrations were determined using the BCA Protein Assay Kit (Invitrogen; Thermo Fisher Scientific, Inc., Waltham, MA, United States). We separated the samples using 10% polyacrylamide gel (40 μg of total protein per lane) and transferred them onto polyvinylidene difluoride membranes. The membranes were blocked with 5% non-fat dried milk and incubated with anti-MBP (MAB386, 1:500, Millipore, Massachusetts, United States) and beta-actin antibodies (ab8227, 1:5,000, Abcam, Cambridge, United Kingdom) overnight at 4°C. We then washed and incubated the membranes with secondary antibodies for 1 h at room temperature. Immunoreactive bands were detected using the Super Signal West Pico Chemiluminescent Substrate (34077; Thermo Fisher Scientific, Inc., Waltham, MA, United States) and visualized on X-ray films. Quantifications were performed using densitometric analysis implemented in the Bio-Rad QuantityOne1-D Analysis Software.

### Statistical Analyses

Results are presented as mean ± standard deviation. The results of the Y-maze test, western blot and characterization of lipid species compositions are analyzed by a one-way analysis of variance and the results of 3-chamber test were analyzed by two-way analysis of variance. The Bonferroni *Post hoc* test was used when appropriate. *P* < 0.05 was deemed statistically significant. Pearson’s correlation coefficient was used to analyze the correlation between the brain lipid species levels and behaviors.

The initial lipidomic data was processed using LipidSearch software 4.1 (Thermo Scientific^TM^, Inc., Waltham, MA, United States) for peak recognition, lipid extraction (secondary appraisal), peak alignment, and quantitative processing. After normalization and integration using the Perato scaling method, the processed data were imported into SIMPCA-P 14.1 (Umetrics, Umea, Sweden) for a multivariate statistical analysis, including a principal component analysis, partial least squares discriminant analysis, and orthogonal partial least squares discriminant analysis. Lipids with significant differences were identified based on a combination of statistically significant thresholds of variable influence on projection values obtained from the orthogonal partial least squares discriminant analysis model (multidimensional analysis) and two-tailed Student’s *t*-test (*P* < 0.05) on the raw data (unidimensional analysis).

## Results

### rTMS Ameliorated CPZ-Induced Behavioral Deficits and Enhanced Remyelination

Previous work already found that CPZ-treated mice showed decreased memory functions and impaired social interaction as assessed by Y-maze test and 3-chamber test (Sen et al., 2019). To determine the effects of rTMS on CPZ-induced cognitive impairment, we carried out the Y-maze and 3-chamber tests 24 h after the final rTMS. In the Y-maze test (Figures 1B–D), significant differences were observed in the actual alternations number [*F*_(2, 33)_ = 5.262, *P* < 0.05] and SA percentage [*F*_(2, 33)_ = 6.626, *P* < 0.01] but not in the total number of arm entries [*F*_(2, 33)_ = 1.534, *P* = 0.2307], the actual alternations number and SA percentage in the CPZ-treated group was less than those in the sham group (CPZ vs. sham, *P* < 0.01), and rTMS significantly ameliorated the CPZ-induced spatial cognitive impairment, as evidenced by the increase in alteration behavior in the treatment group (CPZ + rTMS vs. CPZ, *P* < 0.05) (Figure 1D). In the 3-chamber test, CPZ-treated mice showed suppressed social interaction and social memory compared with mice in sham group, which was ameliorated by 5 Hz rTMS (Figures 1E,F). In session 2, mice in the sham group spent more time in the compartment with subject 1 than in the compartment with the empty-wire cup, while mice in the CPZ group spent a similar amount of time in both chambers. Contrastingly, rTMS resulted in the CPZ-treated mice spending more time in the compartment with subject 1 (Figure 1E). In session 3, when a novel unfamiliar mouse (subject 2) was placed in the previously empty cup, mice in the sham group spent more time with subject 2 than with subject 1, whereas CPZ-treated mice spent much less time with subject 2 than with subject 1. Mice in the CPZ + rTMS group spent more time with subject 2 than with subject 1 (Figure 1F). The levels of myelin basic protein (MBP), indicating the myelination in different brain regions, were detected by western blotting. As shown in Figures 1G–I, significant differences between each group in the expression levels of MBP were observed in the hippocampus [*F*_(2, 9)_ = 35.61, *P* < 0.01], PFC [*F*_(2, 9)_ = 26.39, *P* < 0.01], and striatum [*F*_(2, 9)_ = 20.23, *P* < 0.01]. Compared with mice in the sham group, the CPZ-treated mice had significantly decreased MBP levels in the hippocampus, PFC, and striatum (CPZ vs. sham, *P* < 0.01), while 5 Hz rTMS treatment effectively reversed these changes (CPZ + rTMS vs. CPZ, *P* < 0.01) in PFC and striatum. These results suggest that 5Hz rTMS treatment ameliorated the CPZ-induced cognitive impairment and enhanced remyelination in mice.

### The Impact of rTMS on the Hippocampal Lipidome

**FIGURE 2:**
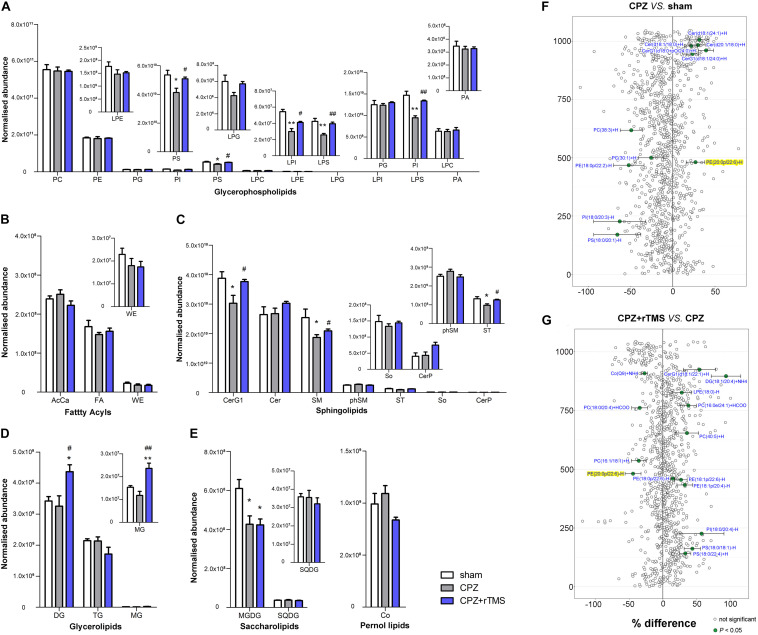
Alteration of lipidomic profiles and the characterization of lipids in the hippocampus of mice in each group. Six main lipid classes were analyzed: **(A)** glycerophospholipids, **(B)** fatty acyls, **(C)** sphingolipids, **(D)** glycerolipids, **(E)** saccharolipids, and pernol lipids. Lipids showing low levels results have been enlarged in insets. **(F,G)** Forest plots of individual lipids in the hippocampus of mice in the CPZ group expressed as a percent difference compared to shams, CPZ + rTMS group expressed as a percent difference compared to the CPZ group. Molecules indicated in green represent significant differences in individual lipids (*P* < 0.05). Lipids highlighted in yellow indicate lipids that were modulated in a contrary direction compared to that in the CPZ group. PC, phosphatidylcholine; PE, phosphatidylethanolamine; PG, phosphatidylglycerol; PI, phosphatidylinositol; PS, phosphatidylserine; LPC, lysophosphatidylcholine; LPE, lysophosphatidylethanolamine; LPG, lysophosphatidylglycerol; LPI, lysophosphatidylinositol; LPS, lysophosphatidylserine; PA, phosphatidic acid; AcCa, acylcarnitine; FA, fatty acid; WE, wax esters; Cer, ceramides; CerG, glucosylceramides; SM, sphingomyelin; phSM, sphingomyelin (phytosphingosine); ST, sulfatides; So, sphingosine; CerP, ceramide phosphates; DG, diglyceride; TG, triglyceride; MG, monoglyceride; MGDG, monogalactosyldiacylglycerol; SQDG, sulfoquinovosyldiacylglycerol; Co, coenzyme. *P < 0.05 vs. sham; **P < 0.01 vs. sham; *^#^P* < 0.05 vs. CPZ; *^##^P* < 0.01 vs. CPZ.

**FIGURE 3:**
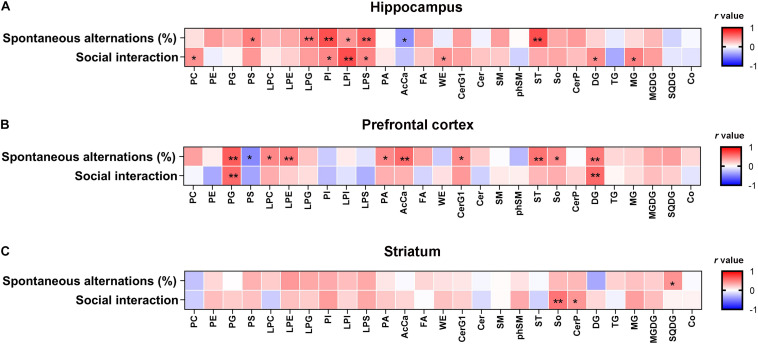
Correlation analysis between cognitive behavior and the lipid levels in the hippocampus, PFC, and striatum. **(A)** The hippocampal PI, PS, LPI, and LPS levels were negatively correlated with the severity of cognitive behavioral impairment. **(B)** The PFC AcCa, DG, and PG levels were negatively correlated with the severity of the cognitive behavioral impairment. **(C)** The striatum SQDG level was positively correlated with the percentage of spontaneous alternations and So levels was positively correlated with the percentage of interaction time. The correlation between the lipid levels and cognitive behavior was analyzed using the Pearson’s correlation coefficient. *P < 0.05, **P < 0.01.

Lipidomic analyses revealed that there were significant differences in the hippocampal concentrations of glycerophospholipids, including phosphatidylserine (PS) [*F*_(2, 21)_ = 5.889, *P* < 0.01], phosphatidylinositol (PI) [*F*_(2, 21)_ = 20.46, *P* < 0.01], lysophosphatidylinositol (LPI) [*F*_(2, 21)_ = 19.49, *P* < 0.01], and phosphatidic acid (LPS) [*F*_(2, 21)_ = 11.12, *P* < 0.01] between each group (Figure 2A). *Post hoc* comparisons revealed that CPZ-treated mice exhibited significantly reduced levels of PS, LPS, PI, and LPI (CPZ vs. sham, *P* < 0.05); these decreased levels were effectively normalized after 5 Hz rTMS (CPZ + rTMS vs. CPZ, *P* < 0.05). Significant differences were also observed in the hippocampal levels of sphingolipids (Figure 2C), glycerolipids (Figure 2D) and saccharolipids (Figure 2E), including glucosylceramides (CerG1) [*F*_(2, 21)_ = 5.079, *P* < 0.05] sphingomyelin (SM) [*F*_(2, 21)_ = 4.708, *P* < 0.05], sulfatide (ST) [*F*_(2, 21)_ = 5.514, *P* < 0.05], diglyceride (DG) [*F*_(2, 21)_ = 5.990, *P* < 0.01], monoglyceride (MG) [*F*_(2, 21)_ = 12.33, *P* < 0.01], and monogalactosyldiacylglycerol (MGDG) [*F*_(2, 21)_ = 6.941, *P* < 0.01]. *Post hoc* comparisons further revealed that CPZ-treated mice exhibited significantly decreased levels of CerG1, SM, ST, and MGDG (CPZ vs. sham, *P* < 0.05); rTMS normalized the levels of CerG1, SM, and ST (CPZ + rTMS vs. CPZ, *P* < 0.05) effectively. Interestingly, rTMS significantly increased the hippocampal levels of DG and MG (Figure 2D). Furthermore, the hippocampal levels of PI (*r* = 0.685, *P* < 0.01), PS (*r* = 0.502, *P* < 0.05), LPI (*r* = 0.426, *P* < 0.05), LPS (*r* = 0.600, *P* < 0.01), and ST (*r* = 0.691, *P* < 0.01) were positively correlated with the SA percentage and the hippocampal levels of AcCa (*r* = –0.439, *P* < 0.05) were negatively correlated with the SA percentage. Additionally, the hippocampal levels of PI (*r* = 0.473, *P* < 0.05), LPI (*r* = 0.696, *P* < 0.01), and LPS (*r* = 0.472, *P* < 0.05) were also positively correlated with the percentage of interaction time (Figure 3A). These results suggest that the hippocampal PI, PS, LPI, and LPS levels were negatively correlated with the severity of cognitive behavioral impairment.

There were also significant differences in the hippocampal lipid profiles across the three groups. Lipidomic analyses revealed that the concentrations of PS(18:0/20:1)−H, PI(18:0/20:3)−H, PE(18:0p/22:2)−H, PC(30:1) + H, and PC(38:3) + H were decreased in the CPZ group compared to those in the sham group, and the levels of PE(20:0p/22:6)−H, CerG1(d18:1/24:0) + H, CerG1(d18:0 + pO/24:0) + H, Cer(d18:1/18:0) + H, Cer(d20:1/18:0) + H, and Cer(d18:1/24:1) + H (Figure 2F) were increased in the CPZ group compared to those in the sham group. We also found that several lipid molecules were markedly changed in the hippocampus of CPZ-treated mice after rTMS; specifically, the concentrations of 15 lipids including PS(18:0/22:4) + H, PS(18:0/18:1)−H, PI(18:0/20:4)−H, PE(18:1p/20:4)−H, PE(18:1p/22:6)−H, PE(18:0p/22:6)−H, PC(40:5) + H, PC(16:0e/24:1) + HCOO, LPE(18:0)−H, DG(18:1/20:4) + NH4, and CerG1(d18:1/22:1) + H were increased, and the relative abundances of Co(Q9) + NH4, PC(18:0/20:4) + HCOO, PC(16:1/18:1) + H, and PE(20:0p/22:6)−H were decreased in the hippocampus of CPZ-treated mice after rTMS (Figure 2G). Taken together, glycerophospholipids and sphingolipids were the predominantly disturbed lipids in the hippocampus after CPZ treatment, and they were partially restored after rTMS.

### The Impact of rTMS on the PFC Lipidome

**FIGURE 4:**
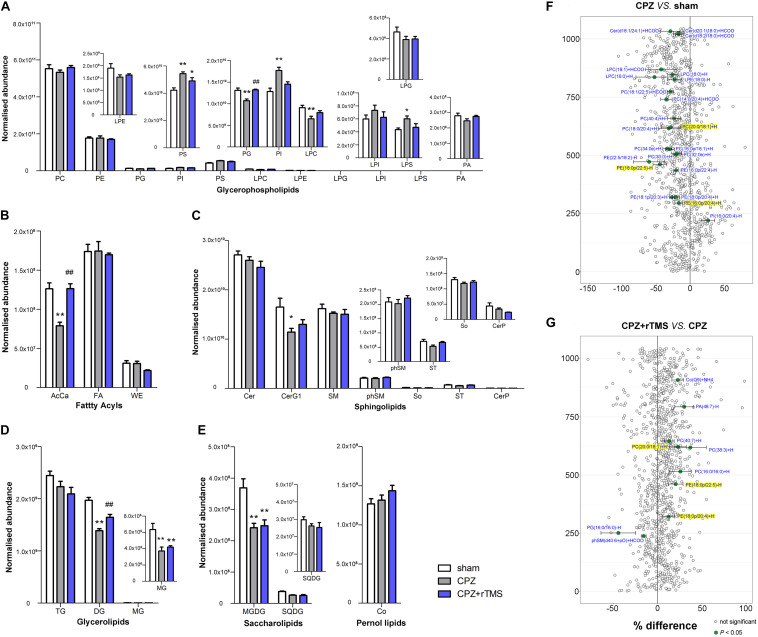
Alterations in lipidomic profiles and the characterization of lipids in the PFC of mice in each group. Six main lipid classes were analyzed: **(A)** glycerophospholipids, **(B)** fatty acyls, **(C)** sphingolipids, **(D)** glycerolipids, **(E)** saccharolipids, and pernol lipids. Lipids showing low levels results have been enlarged in insets. **(F,G)** Forest plots of individual lipids in the PFC of mice in the CPZ group expressed as a percent difference compared to shams, CPZ + rTMS group expressed as a percent difference compared to the CPZ group. Molecules indicated in green represent significant differences in individual lipids (*P* < 0.05). Lipids highlighted in yellow indicate lipids that were modulated in a contrary direction compared to that in the CPZ group. PC, phosphatidylcholine; PE, phosphatidylethanolamine; PG, phosphatidylglycerol; PI, phosphatidylinositol; PS, phosphatidylserine; LPC, lysophosphatidylcholine; LPE, lysophosphatidylethanolamine; LPG, lysophosphatidylglycerol; LPI, lysophosphatidylinositol; LPS, lysophosphatidylserine; PA, phosphatidic acid; AcCa, acylcarnitine; FA, fatty acid; WE, wax esters; Cer, ceramides; CerG, glucosylceramides; SM, sphingomyelin; phSM, sphingomyelin (phytosphingosine); ST, sulfatides; So, sphingosine; CerP, ceramide phosphates; DG, diglyceride; TG, triglyceride; MG, monoglyceride; MGDG, monogalactosyldiacylglycerol; SQDG, sulfoquinovosyldiacylglycerol; Co, coenzyme. *P < 0.05 vs. sham; **P < 0.01 vs. sham; *^##^P* < 0.01 vs. CPZ.

Significant differences were observed in the levels of glycerophospholipids, including PS [*F*_(2, 21)_ = 10.74, *P* < 0.01], PG [*F*_(2, 21)_ = 12.66, *P* < 0.01], LPC [*F*_(2, 21)_ = 6.078, *P* < 0.01], PI [*F*_(2, 21)_ = 12.22, *P* < 0.01], and LPS [*F*_(2, 21)_ = 3.907, *P* < 0.05] between each group in the PFC (Figure 4A). *Post hoc* comparisons indicated that the levels of PI, PS, and LPS (CPZ vs. sham, *P* < 0.05) were increased, but those of PG and LPC were decreased after CPZ treatment (CPZ vs. sham, *P* < 0.01). rTMS effectively normalized the levels of PG in the PFC (CPZ + rTMS vs. CPZ, *P* < 0.05), but not the levels of PS, PI, LPC, and LPS in the PFC (CPZ + rTMS vs. CPZ, *P* > 0.05) (Figure4A). There were also significant differences in the levels of fatty acyls, sphingolipids, glycerolipids, and saccharolipids, including AcCa [*F*_(2, 21)_ = 19.71, *P* < 0.01], CerG1 [*F*_(2, 21)_ = 4.306, *P* < 0.05], DG [*F*_(2, 21)_ = 33.61, *P* < 0.01], MG [*F*_(2, 21)_ = 15.25, *P* < 0.01], MGDG [*F*_(2, 21)_ = 11.11, *P* < 0.01], and SQDG [*F*_(2, 21)_ = 11.70, *P* < 0.01] in the PFC (Figures 4B–E). *Post hoc* comparisons further revealed that CPZ administration significantly decreased the levels of AcCa, DG, MG, MGDG, and CerG1 (CPZ vs. sham, *P* < 0.01) in the PFC; rTMS only normalized the levels of AcCa and DG in the PFC (CPZ + rTMS vs. CPZ, *P* < 0.01) of CPZ-treated mice. Furthermore, the levels of AcCa (*r* = 0.581, *P* < 0.01), DG (*r* = 0.523, *P* < 0.01), and PG (*r* = 0.637, *P* < 0.01) were positively correlated with the SA percentage, while those of DG (*r* = 0.590, *P* < 0.01) and PG (*r* = 0.589, *P* < 0.01) were also positively correlated with the percentage of interaction time (Figure 3B). This suggests that the relative levels of AcCa, DG, and PG in the PFC were negatively correlated with the severity of the cognitive behavioral impairment.

Lipidomic analysis in the PFC revealed changes in lipid concentrations from multiple classes following CPZ administration and rTMS. In general, concentrations of 22 lipids including PE(16:0p/20:4) + H, PE(18:1p/20:3) + H, PE(18:0p/20:4) + H, PE(16:0p/22:4)−H, PE(18:0p/22:5)−H, PE(22:5/18:2) −H, PC(30:0) + H, PC(32:0e) + H, PC(16:0e/18:1) + H, PC(34:0e) + H, PC(18:0/20:4) + H, PC(20:0/18:1) + H, PC(40:4) + H, PC(14:0/20:4) + HCOO, PC(18:1/22:5) + HCOO, LPE(18:0) − H, LPC(16:0) + H, LPC(18:0) + H, LPC(18:1) + HCOO, Cer(d18:2/18:0) + HCOO, Cer(d20:1/18:0) + HCOO, and Cer(d18:1/24:1) + HCOO were decreased, but those of PE(18:0/16:0) were increased in the PFC of CPZ-treated mice compared to those in the PFC of mice in the sham group (Figure 4F). rTMS increased the levels of eight lipids including PE(18:0p/20:4) + H, PE(18:0p/22:5) − H, PC(16:0/16:0) + H, PC(38:3) + H, PC(20:0/18:1) + H, PC(40:7) + H, PA(48:7) − H, and Co(Q9) + NH4, but decreased the levels of phSM(d40:6 + pO) + HCOO and PG(16:0/16:0) − H in the PFC of CPZ-treated mice (Figure 4G). These results indicate that CPZ administration caused a series of changes in PFC lipids, which were partially restored by rTMS.

### The Impact of rTMS on the Striatum Lipidome

**FIGURE 5:**
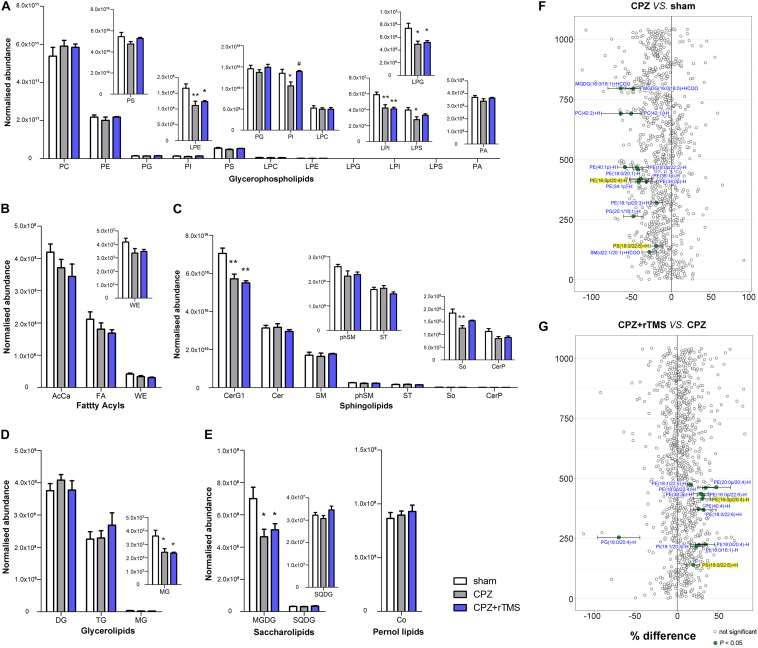
Alterations in lipidomic profiles and the characterization of lipids in the striatum of mice in each group. Six main lipid classes were analyzed: **(A)** glycerophospholipids, **(B)** fatty acyls, **(C)** sphingolipids, **(D)** glycerolipids, **(E)** saccharolipids, and pernol lipids. Lipids showing low levels results have been enlarged in insets. **(F,G)** Forest plots of individual lipids in the striatum of mice in the CPZ group expressed as a percent difference compared to shams, CPZ + rTMS group expressed as a percent difference compared to the CPZ group. Molecules indicated in green represent significant differences in individual lipids (*P* < 0.05). Lipids highlighted in yellow indicate lipids that were modulated in a contrary direction compared to the CPZ group. PC, phosphatidylcholine; PE, phosphatidylethanolamine; PG, phosphatidylglycerol; PI, phosphatidylinositol; PS, phosphatidylserine; LPC, lysophosphatidylcholine; LPE, lysophosphatidylethanolamine; LPG, lysophosphatidylglycerol; LPI, lysophosphatidylinositol; LPS, lysophosphatidylserine; PA, phosphatidic acid; AcCa, acylcarnitine; FA, fatty acid; WE, wax esters; Cer, ceramides; CerG, glucosylceramides; SM, sphingomyelin; phSM, sphingomyelin (phytosphingosine); ST, sulfatides; So, sphingosine; CerP, ceramide phosphates; DG, diglyceride; TG, triglyceride; MG, monoglyceride; MGDG, monogalactosyldiacylglycerol; SQDG, sulfoquinovosyl diacylglycerol; Co, coenzyme. *P < 0.05 vs. sham; **P < 0.01 vs. sham; *^#^P* < 0.05 vs. CPZ.

As shown in Figure 5, there were also significant differences in the striatal levels of PI [*F*_(2, 21)_ = 5.645, *P* < 0.05], LPI [*F*_(2, 21)_ = 8.928, *P* < 0.01], LPE [*F*_(2, 21)_ = 6.504, *P* < 0.01], LPG [*F*_(2, 21)_ = 5.846, *P* < 0.01], LPS [*F*_(2, 21)_ = 4.358, *P* < 0.05], CerG1 [*F*_(2, 21)_ = 13.48, *P* < 0.01], So [*F*_(2, 21)_ = 7.410, *P* < 0.01], MG [*F*_(2, 21)_ = 5.799, *P* < 0.01], and MGDG [*F*_(2, 21)_ = 5.503, *P* < 0.05] between each group. *Post hoc* comparisons indicated that the striatal levels of LPI, LPE, CerG1, LPG, PI, LPS, So, MG, and MGDG (CPZ vs. sham, *P* < 0.05) were decreased in CPZ-treated mice. However, rTMS treatment only normalized the striatal levels of PI effectively in CPZ-treated mice (CPZ + rTMS vs. CPZ, *P* < 0.05). Additionally, we found that striatal levels of 15 lipids including SM(d22:1/20:1) + HCOO, PS(18:0/22:6) + H, PG(20:1/18:1)−H, PE(18:1p/20:3) + H, PE(34:1p)−H, PE(34:0p)−H, PE(16:0p/20:4) −H, PE(36:1p)−H, PE(18:0/20:1)−H, PE(18:0p/22:2)−H, PE(40:1p)−H, PC(42:2) + H, PC(42:1) + H, MGDG(16:0/18:0) + HCOO, and MGDG(18:0/18:1) + HCOO were markedly decreased in CPZ-treated mice compared to mice in the sham group (Figure 5F). Moreover, the striatal concentration of PG(16:0/20:4)−H was decreased, while those of PS(18:0/22:6) + H, PI(18:0/16:1)−H, PI(18:1/20:4)−H, PI(18:0/20:4)−H, PE(18:0/22:6) + H, PE(40:4) + H, PE(16:0p/20:4)−H, PE(16:0p/22:6)−H, PE(38:3p)−H, PE(18:0p/22:4)−H, PE(20:0p/20:4)−H and PE(18:1/22:5)−H were increased in CPZ-treated mice after rTMS (Figure 5G). Furthermore, the striatal SQDG (*r* = 0.425, *P* < 0.05) levels were positively correlated with the SA percentage, and So (*r* = 0.542, *P* < 0.01) levels were positively correlated with the percentage of interaction time (Figure 3C).

## Discussion

In this study, we performed a lipidomic analysis to assess the effect of 5 Hz rTMS on the lipidome of the hippocampus, PFC, and striatum in a CPZ-induced mice model of demyelination. CPZ treatment induced cognitive impairment and remarkable changes in brain lipids, specifically in glycerophospholipids. Moreover, the changes in lipids of the PFC induced by CPZ were more extensive compared to those observed in the hippocampus and striatum. Notably, rTMS ameliorated CPZ-induced cognitive impairment and partly normalized CPZ-induced lipid changes, especially in the hippocampus. Thus, the region-specific regulation of the brain lipidome may be partly involved in the therapeutic effects of rTMS.

Lipids are important in maintaining and regulating brain development and function (Glade and Smith, 2015; Lauritzen et al., 2016; Hussain et al., 2019). Myelin is composed primarily of lipids, and the modulation of lipid homeostasis is a potential target for the improvement of psychiatric symptoms (Hamazaki et al., 2015; Kavoor et al., 2017). Membrane lipids, which are predominantly composed of glycerophospholipids, sphingolipids, and cholesterol, are crucial structural components of the brain (Xue et al., 2020). Glycerophospholipids are major components of myelin and essential for normal cognitive function (Blusztajn et al., 2017; Lee et al., 2017). The typical glycerophospholipids found in mammalian membranes are PC, PE, PS, PA, and PI. PC is the most abundant phospholipid in mammalian, plant, and yeast cells, whereas PS and PE are two metabolically related aminophospholipids in the membranes of all eukaryotic and prokaryotic cells; PS undergoes decarboxylation via the mitochondrial enzyme PS decarboxylase to give PE (Vance and Tasseva, 2013). The levels of PS and PC have been found to be increased in the left thalamus of patients with schizophrenia (Schmitt et al., 2004). PC, LPC, and LPE were downregulated in the plasma of patients with schizophrenia before treatment with antipsychotics, and PC and LPC were further downregulated after treatment (Yan et al., 2018), indicating that phospholipids might play a role in the etiology of schizophrenia. Moreover, a recent study found that the concentration of PC was reduced along with CPZ-induced demyelination in the corpus callosum of mice and human post-mortem brains (Trepanier et al., 2018). In this study, we found that modulation of glycerophospholipid levels by rTMS in CPZ-treated mice brains was region-specific. CPZ led to a significant decrease in the levels of PI, LPI, PS, and LPS in the hippocampus, PG and LPC in the PFC, and LPI, LPE, LPG, PI, and LPS in the striatum. However, CPZ also led to a significant increase in the levels of PI, PS, and LPS in the PFC. Notably, alterations in the levels of glycerophospholipids in the hippocampus, PG, PI, LPC, and LPS in the PFC, and PI and LPS in the striatum of CPZ-treated mice were normalized by rTMS (Figures 2, 4, 5A–E), indicating that rTMS affects the composition of glycerophospholipids, particularly PI and LPS, in the brain. Our lipidomic analyses further revealed that the PE(20:0p/22:6)−H levels were increased in the hippocampus, PE(18:0p/20:4) + H, PE(18:0p/22:5)−H, and PC(20:0/18:1) + H levels were decreased in the PFC, and PE(16:0p/20:4)−H and PS(18:0/22:6) + H levels were decreased in the striatum of CPZ-treated mice, all of which were normalized by rTMS (Figures 2, 4, 5F,G). Taken together, these results indicate that glycerophospholipids are a potential target of rTMS. The mechanisms of the therapeutic effects of rTMS have been elucidated in literature. It has been indicated previously that rTMS could induce changes in the cerebral blood flow (Knoch et al., 2006; Shang et al., 2018), release of neurotransmitters and brain-derived neurotrophic factor (Lu et al., 2018; Peng et al., 2018; Feng et al., 2019), and synaptic plasticity over the stimulated area (Cirillo et al., 2017). We previously found that the regulation of endocannabinoid signaling was involved in the antidepressant effects of rTMS (Fang and Wang, 2018); notably, the biosynthesis of endogenous cannabinoids depends on several glycerophospholipids, such as PE, PC, PA, and PI (Malcher-Lopes et al., 2008). Thus, the regulation of glycerophospholipids might be involved in the therapeutic effects of rTMS.

Sphingolipids are also major components of myelin. SM constitutes the vast majority of cellular sphingolipids; it is particularly abundant in the myelin sheath that surrounds neuronal axons and plays an important role in myelin integrity and function as well as cognitive development (Don et al., 2014; Schneider et al., 2019). Our findings also showed that CPZ treatment decreased the levels of SM, CerG1, and ST in the hippocampus, CerG1 in the PFC, and CerG1 and So in the striatum, suggesting that the modulation of sphingolipid levels in the brain by CPZ was region-specific. However, rTMS only normalized the levels of sphingolipids in the hippocampus and PFC. Furthermore, a growing body of evidence indicates that abnormalities in glycerolipid and fatty acyl compositions may also be involved in demyelination (Sanchez et al., 1998; Pottie et al., 2011). The present study showed that CPZ treatment decreased glycerolipid levels in both the PFC (DG and MG) and striatum (MG). However, the reductions of MG in both PFC and striatum were not normalized by rTMS, suggesting that the glycerolipids in the PFC and striatum are more vulnerable than hippocampus to CPZ. AcCa compounds in the brain functionally alter and stabilize membranes, improving mitochondrial function and increasing antioxidant activity (Rutkowsky et al., 2014; Tarasenko et al., 2018). The present study also found that CPZ treatment decreased the levels of AcCa only in the PFC, which was normalized by rTMS, suggesting that the AcCa in the PFC are more vulnerable than other brain region and might be associated to the therapeutic effects of rTMS. Notably, the levels of glycerolipids in hippocampus were not changed in CPZ-treated mice. However, rTMS treatment significantly increased the levels of DG and MG. Furthermore, CPZ reduced the levels of MGDG, and this was not normalized by rTMS. These results suggest that the modulation of brain glycerolipids by rTMS is region-specific and the decreased MGDG levels might be associated with residual symptoms of CPZ-induced demyelination.

The biological effects of rTMS are dependent on the parameters and duration of treatment. Recently, we reported that rTMS treatment for 7 consecutive days (5Hz, 1.26 Tesla) ameliorated depressive-like behaviors in chronic unpredictable stress-treated rats (Xue et al., 2019). Here, we conducted lipidomic profiling on 1189 lipid species from 29 classes in the hippocampus, PFC, and striatum of mice treated with rTMS for 7 days after a 6-weeks CPZ paradigm. This thorough analysis of the brain lipidome reveals the PFC as a key target of CPZ-induced demyelination, with the highest degree of molecular changes in brain lipids induced by CPZ. The PFC is a key brain region implicated in numerous neuropsychiatric disorders, including schizophrenia (Giraldo-Chica et al., 2018), MDD (Seo et al., 2017), and Alzheimer’s disease (Aarons et al., 2019). Moreover, the PFC also exhibits later myelination during early neonatal life, making it more susceptible to external influences (Hoban et al., 2016). This region-specific vulnerability corroborates the findings of previous studies showing major lipid alterations in the PFC after stress (Oliveira et al., 2016). However, since we observed just cognitive behavior in this study, the association between abnormal lipid levels and emotion-related behaviors after rTMS remains unclear. Moreover, we investigated neither the effects of rTMS at different time points, nor the intensity and frequency-response relationships between rTMS and the composition of brain lipids. Finally, we were unable to rule out the effects of rTMS on other areas than the prefrontal cortex because there were no coils specifically for mice. In the future, the time- and dose- effects as well as different parameters of rTMS on the composition of brain lipids needs to be further investigated. Additionally, the precise mechanism by which rTMS regulates lipid composition also requires further investigation.

## Conclusion

In summary, our study revealed distinct changes in the lipid composition within the hippocampus, PFC, and striatum in CPZ-treated mice. We further confirmed that the PFC is relatively more sensitive than the hippocampus and striatum to CPZ-induced lipid modulation and showed that rTMS is effective in normalizing the changes in lipid composition induced by CPZ, especially glycerophospholipids. Thus, the regulation of the brain lipidome may be involved in the therapeutic effects of rTMS. Further studies are required to explore the more detailed signaling cascades of rTMS, including the effects of rTMS at different time points, the intensity and frequency-response relationships between rTMS and the composition of brain lipids, the influence of rTMS on healthy brain lipids, as well as the precise mechanism through which rTMS regulates lipid composition.

## Data Availability Statement

The original contributions presented in the study are included in the article/supplementary material, further inquiries can be directed to the corresponding author/s.

## Ethics Statement

The animal study was reviewed and approved by Animal Use and Protection Committee of the Fourth Military Medical University. Written informed consent was obtained from the owners for the participation of their animals in this study.

## Author Contributions

ZP, QT, and HW were involved in conception and design of the study. CZ, MC, YW, WW, YY, XW, and GH conducted the final data and wrote the manuscript. All authors discussed and edited the manuscript.

## Conflict of Interest

The authors declare that the research was conducted in the absence of any commercial or financial relationships that could be construed as a potential conflict of interest.

## Abbreviations

rTMS: repetitive transcranial magnetic stimulation
CPZ: cuprizone
PFC: prefrontal cortex
MD: major depression
PTSD: posttraumatic stress disorder
CNS: central nervous system
MTBE: methyl tert-butyl ether
SD: standard deviation
MBP: myelin basic protein
ANOVA: one-way analysis of variance
PCA: principal component analysis
PLS-DA: partial least squares discriminant analysis
OPLS-DA: orthogonal partial least squares discriminant analysis
VIP: variable influence on projection
LPI: lysophosphatidylinositol
PI: phosphatidylinositol
PS: phosphatidylserine
LPS: phosphatidic acid
PA: phosphatidic acid
PE: phosphatidylinositol
LPC: lysophosphatidylcholine
PC: phosphatidylcholine
PG: phosphatidylglycerol
LPG: lysophosphatidylglycerol
AcCa: acylcarnitine
Cer: ceramides
CerG1: glucosylceramides
SM: sphingomyelin
phSM: sphingomyelin (phytosphingosine)
ST: sulfatides
So: sphingosine
CerP: ceramide phosphate
ST: sulfatide
DG: diglyceride
TG: triglyceride
MG: monoglyceride
SQDG: sulfoquinovosyldiacylglycerol
MGDG: monogalactosyldiacylglycerol
Co: coenzyme
PLD: phospholipase D
CL: cardiolipin.
